# Bone morphogenetic protein 4 meets artificial intelligence: a comprehensive review of AI-driven advances in drug discovery, structural modeling, and clinical translation

**DOI:** 10.3389/fmolb.2026.1930582

**Published:** 2026-10-20

**Authors:** Wanli Sun, Tongtong Huo, Zhewei Ye, Wu Wei, Yanqing Sun, Hui Cai

**Affiliations:** 1 School of Traditional Chinese and Western Medicine, Gansu University of Chinese Medicine, Lanzhou, Gansu, China; 2 Gansu Provincial Hospital, Lanzhou, Gansu, China; 3 Wuhan University of Science and Technology, Wuhan, China; 4 Tongji Medical College, Union Hospital, Huazhong University of Science and Technology, Wuhan, China; 5 NHC Key Laboratory of Diagnosis and Therapy of Gastrointestinal Tumor, Gansu Provincial Hospital, Lanzhou, Gansu, China; 6 Key Laboratory of Molecular Diagnostics and Precision Medicine for Surgical Oncology in Gansu Province, Gansu Provincial Hospital, Lanzhou, Gansu, China

**Keywords:** AlphaFold, artificial intelligence, BMP4, drug discovery, machine learning, protein structure prediction

## Abstract

**Background:**

Bone Morphogenetic Protein 4 (BMP4), a member of the transforming growth factor-beta (TGF-β) superfamily, plays pivotal roles in embryonic development, tissue homeostasis, and disease pathogenesis—including cancer, fibrosis, and fibrodysplasia ossificans progressiva (FOP). The rapid advancement of artificial intelligence (AI) and machine learning (ML) methodologies over the past 5 years has created unprecedented opportunities for understanding and therapeutically targeting BMP4 biology at multiple scales, from atomic-resolution structure prediction to population-level clinical prognostication.

**Objective:**

This review systematically examines the intersection of BMP4 biology and AI across three core domains: (1) AI-driven drug discovery targeting BMP4 signaling pathways, (2) deep learning-based protein structure prediction and computational modeling of BMP4 and its receptor complexes, and (3) clinical AI applications for BMP4-associated diseases.

**Methods:**

A narrative review with systematic search elements was conducted across PubMed, Scopus, Web of Science, bioRxiv, and arXiv for English-language publications from 2020 to 2026, supplemented by targeted searches via Semantic Scholar. Included studies were categorized as BMP4-specific, BMP receptor/BMP-pathway-targeted, or transferable AI methodology not yet applied to BMP4.

**Results:**

We find that AI has accelerated BMP4-related research along multiple axes, though with important caveats. AlphaFold3 and related tools have generated high-confidence predicted models of BMP4-receptor complex architecture, aspects of which were previously intractable to experimental structure determination; these predictions remain to be fully validated experimentally. ML-guided virtual screening has identified first-in-class BMP pathway (receptor) modulators for FOP, although BMP4-specific small-molecule modulators have not yet been identified computationally. Single-cell transcriptomics combined with machine learning have revealed BMP4’s context-dependent roles in glioblastoma cell state transitions and cancer-associated fibroblast biology; and LASSO-Cox regression models incorporating BMP4 expression have shown promising prognostic value in pancreatic adenocarcinoma and gastric cancer, pending prospective validation. Significant gaps persist—most notably the absence of BMP4-specific *de novo* protein design efforts, the scarcity of large-scale training datasets for BMP4-targeted drug discovery, and the limited integration of multimodal AI approaches in BMP4 clinical research.

**Conclusion:**

The convergence of BMP4 biology and AI represents a fertile research Frontier. While foundational tools are now in place, realizing the full translational potential of AI-augmented BMP4 research will require coordinated efforts in data generation, model development, and rigorous experimental and clinical validation. We outline priority directions for the next 5 years (approximately 2026–2031).

## 1 Introduction

### 1.1 BMP4 biology and signaling

Bone Morphogenetic Protein 4 (BMP4) is a secreted morphogen of the transforming growth factor-beta (TGF-β) superfamily that governs a remarkably diverse repertoire of biological processes, including mesoderm specification, neurogenesis, limb development, and tissue homeostasis (Wang et al., 2014). Understanding how BMP4 signal is transduced from the extracellular space to the nucleus is essential background for the AI applications discussed throughout this review, and is summarized in Figure 1.

**FIGURE 1:**
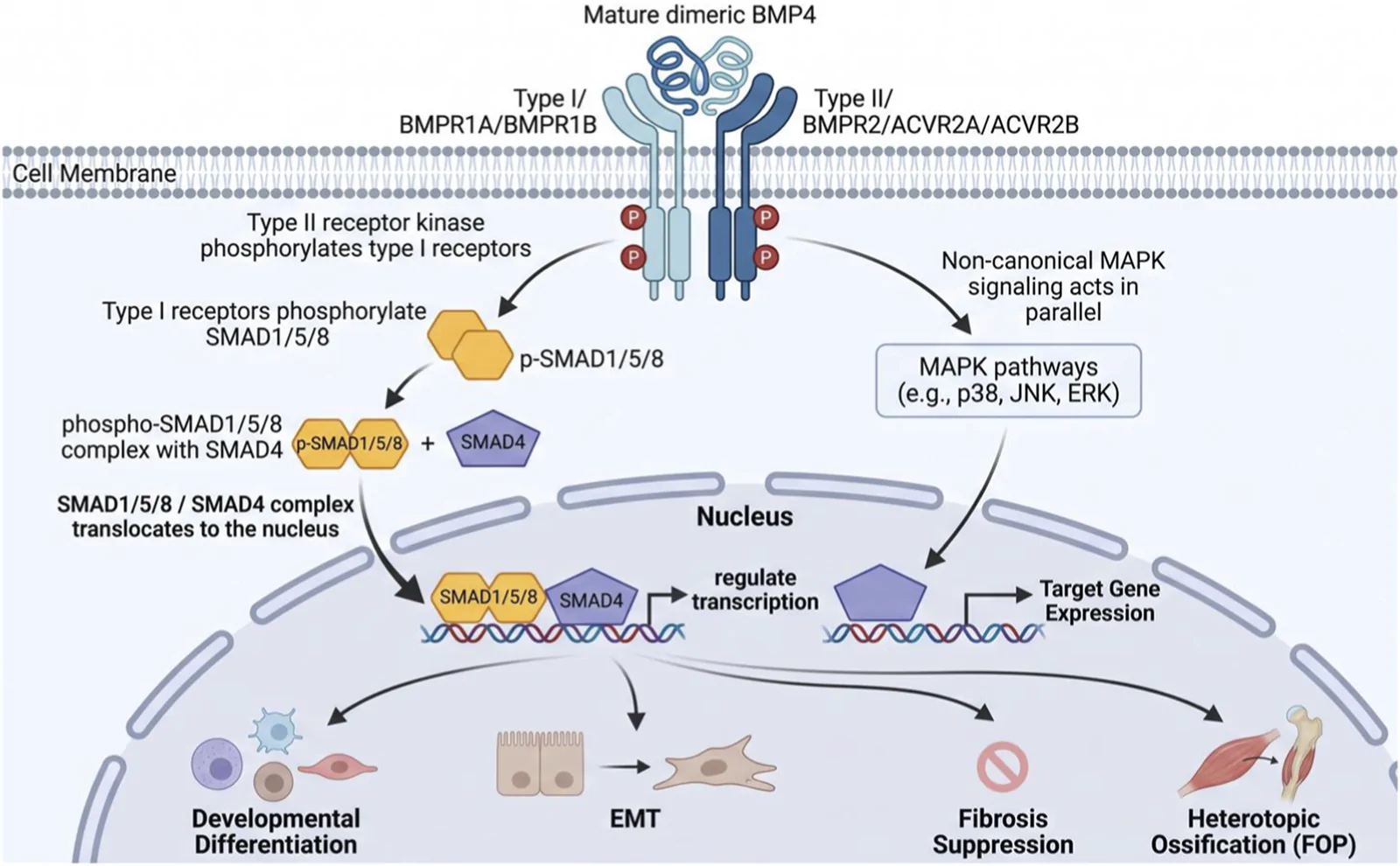
BMP4 signaling pathway overview. Mature dimeric BMP4 binds the type I/type II receptor heterotetramer (BMPR1A/BMPR1B, BMPR2/ACVR2A/ACVR2B); the type II receptor kinase phosphorylates type I receptors, which phosphorylate SMAD1/5/8; phospho-SMAD1/5/8 complex with SMAD4 and translocate to the nucleus to regulate transcription. Non-canonical MAPK signaling acts in parallel. Downstream, context-dependent outcomes range from developmental differentiation to EMT, fibrosis suppression, and heterotopic ossification (FOP).

In brief, mature dimeric BMP4 initiates signaling by binding to a heterotetrameric receptor complex comprising two type I (BMPR1A/ALK3, BMPR1B/ALK6) and two type II (BMPR2, ACVR2A, ACVR2B) serine/threonine kinase receptors (Miyazono et al., 2010). Ligand binding induces the constitutively active type II receptor kinases to phosphorylate the type I receptors, which in turn phosphorylate the receptor-regulated SMAD proteins SMAD1/5/8. Phosphorylated SMAD1/5/8 form heteromeric complexes with the common-mediator SMAD4, and these complexes translocate to the nucleus, where—together with cell-type-specific co-factors—they regulate the transcription of target genes (Miyazono et al., 2010). In parallel, BMP4 activates non-canonical mitogen-activated protein kinase (MAPK) pathways. The downstream biological outcome is profoundly context dependent: BMP4 signaling can drive mesodermal differentiation during development, promote epithelial-mesenchymal transition (EMT) and tumor progression in some cancers, suppress fibrosis in the lung, or—when amplified by gain-of-function mutations in the type I receptor ACVR1/ALK2 — drive heterotopic ossification in FOP (Figure 1) (Wang et al., 2014; Miyazono et al., 2010).

The dysregulation of BMP4 signaling has been causally implicated in a spectrum of human diseases: its overexpression drives EMT and poor prognosis in pancreatic and gastric cancers (Deng et al., 2023); its loss contributes to pulmonary fibrosis (Guan et al., 2022); and its hyperactive downstream signaling—mediated by gain-of-function mutations in the type I receptor ACVR1/ALK2 — underlies the catastrophic heterotopic ossification of FOP (Kaplan et al., 2025).

### 1.2 Rationale for an AI-centered focus

The selection of BMP4 as the primary focus rests on two core dimensions: 1) BMP4 exhibits extreme context dependence. It acts as an oncogenic driver in pancreatic and gastric cancers, plays variable roles in glioblastoma, functions as a tumor suppressor in other environments, and serves as an anti-fibrotic factor in pulmonary fibrosis (Deng et al., 2023; Guan et al., 2022). Its signaling architecture is highly complex; synthesized as an inactive proprotein, it requires furin-mediated cleavage, is regulated by a prodomain, and transduces signals through multiple type I/type II receptor combinations via both SMAD-dependent and independent networks. This intricate network and functional duality render conventional agonism or antagonism high-risk, mandating AI-driven design for tissue-specific control, and 2) BMP4 data distribution demonstrates a severe structural imbalance. At the systems biology level, BMP4 has accumulated large-scale, comprehensive datasets across multiple disease types, encompassing single-cell transcriptomic, bulk transcriptomic, structural, and clinical data. At the molecular pharmacology level, known BMP4-specific small-molecule modulators and corresponding AI-targeted studies are extremely scarce. This juxtaposition—abundant multi-omics data paired with a critical deficit in targeted small-molecule data—establishes BMP4 as a standardized test case for validating small-sample machine learning methods and explicitly defines the current core technological bottleneck in the field.

### 1.3 The AI revolution in biomedicine

Over the past 5 years, artificial intelligence (AI) has undergone a parallel revolution in the biomedical sciences. The 2024 Nobel Prize in Chemistry, awarded to David Baker for computational protein design, and to Demis Hassabis and John Jumper for protein structure prediction, epitomized the transformative impact of deep learning on structural biology (Jumper et al., 2021). Beyond protein structure prediction, AI methodologies—ranging from graph neural networks and transformer architectures to random forest classifiers and LASSO-Cox regression—have permeated virtually every stage of biomedical research, from early-stage drug discovery to clinical decision support (Table 1). Key AI and computational terms used in this review are defined in the Glossary (Box 1) for readers without a background in these methods.

**TABLE 1:** Summary of representative AI/ML methodologies and their BMP4 evidence level**.**

Key citations	Methodology	Application	BMP4 evidence level
(Tyagi et al., 2023)	Data mining, structure-based virtual screening, docking, 100-ns MD	Screened STITCH/PubChem to identify Retinoic acid and Torularhodin as candidate BMP4 activators	BMP4-specific (computational; not AI/ML)
(Yang et al., 2025)	Structure-based *in silico* screening + SMAD1 phosphorylation assays	RK783, a selective BMP type I receptor (ACVR1) kinase inhibitor for FOP	BMP receptor/BMP-pathway
(Kong et al., 2024)	Naive Bayes with ECFP6 fingerprints	Ligand-based screening with ∼200 actives (ULK1)	Transferable (not yet applied to BMP4)
(Hossain et al., 2025)	Neuro-symbolic AI (logic tensor networks + ChemBERTa)	DPP-4 inhibitor classification (97.25%)	Transferable
(Morehead and Cheng, 2024); (Srinivasan et al., 2026)	3D diffusion; GPT-4o + inductive logic programming	*De novo* generation of synthesizable leads	Transferable
(Abramson et al., 2024); (Bertoline et al., 2023)	AlphaFold3 diffusion architecture (multi-entity + PTMs)	Modeled BMP4 prodomain phosphorylation; furin-cleavage accessibility	BMP4-specific (prediction + experimental mutagenesis)
(Lin et al., 2023)	Protein language model (ESMFold), MSA-free	Millisecond-speed structure prediction for low-MSA targets	Transferable (not yet BMP4-specific)
(Shaikh et al., 2024)	Multi-objective Pareto optimization of BMP/SMAD network	Provided evidence the BMP pathway operates near a Pareto front	BMP receptor/BMP-pathway
(Wang et al., 2025)	scRNA-seq + bulk + prognostic nomogram	BMP4 in PAAD; metabolic (non-canonical) regulation; prognostic utility	BMP4-specific
(Xiao et al., 2025)	Random Forest; DeepSurv	PDAC diagnosis (AUC 0.889) and survival prediction	Disease-associated (BMP4 in panel; not BMP4-specific)
(Verploegh et al., 2022)	RNA-velocity trajectory analysis	BMP4 context-dependent GBM cell state transitions	BMP4-specific
(Larsson et al., 2021)	STAG mathematical model (lineage tracing)	BMP4 shifts GBM stem-like/invasive equilibrium	Disease-associated (BMP4-modulated)
(Bumbaca et al., 2025)	snRNA-seq deconvolution + phosphoproteomics + MaBoSS Boolean networks + ML	GBM cell-state transition prediction	Disease-associated
(Deng et al., 2023)	scRNA-seq + bulk + LASSO-Cox + TIDE	5-gene hypoxia-immune panel incl. BMP4 (gastric cancer)	Disease-associated (BMP4 in panel)
(Ghandikota et al., 2022); (Bayati et al., 2023)	WGCNA; PPI hub-gene extraction	BMP signaling enrichment in IPF modules; Bmp4 downregulation	BMP receptor/BMP-pathway
(Watson et al., 2023); (Dauparas et al., 2022); (Theodoris et al., 2023); (Cui et al., 2024)	RFdiffusion; ProteinMPNN; single-cell foundation models	*De novo* design and perturbation forecasting	Unexplored for BMP4 (knowledge gap)

Abbreviations: AUC, area under the receiver operating characteristic curve; DL, deep learning; GBM, glioblastoma multiforme; IPF, idiopathic pulmonary fibrosis; LASSO, least absolute shrinkage and selection operator; MaBoSS, markovian boolean stochastic simulator; MD, molecular dynamics; PAAD, pancreatic adenocarcinoma; PDAC, pancreatic ductal adenocarcinoma; sc = single cell; STAG, state transition and growth; TIDE, tumor immune dysfunction and exclusion; WGCNA, weighted gene co-expression network analysis.

The convergence of these two trajectories has created a fertile, albeit nascent, research Frontier. Yet, to our knowledge, no comprehensive review has systematically mapped the landscape at this intersection. This article addresses that gap by providing a structured synthesis of AI applications across three domains of BMP4 research: drug discovery, structural and computational modeling, and clinical translation. We conclude by identifying critical knowledge gaps and proposing priority directions for future investigation.

## 2 Methods: literature search strategy

This is a narrative review with systematic search elements; it does not perform quantitative synthesis and is therefore not reported according to PRISMA guidelines. We searched PubMed, Scopus, Web of Science, bioRxiv, and arXiv for English-language publications from 1 January 2020 to 1 July 2026 (the “past 5 years” referenced in the Abstract and Conclusion), supplemented by targeted searches via Semantic Scholar.

Representative Boolean queries included (“BMP4″ OR “bone morphogenetic protein 4″) AND (“artificial intelligence” OR “machine learning” OR “deep learning” OR “AlphaFold” OR “neural network” OR “virtual screening”) (“BMP4″ OR “bone morphogenetic protein 4″) AND (“single-cell” OR “scRNA-seq” OR “multi-omics” OR “prognos*”) (“BMP4″ OR “bone morphogenetic protein 4″) AND (“pancreatic” OR “gastric” OR “glioblastoma” OR “pulmonary fibrosis” OR “fibrodysplasia ossificans progressiva”) AND (“machine learning” OR “deep learning” OR “artificial intelligence”), and (“RFdiffusion” OR “ProteinMPNN” OR “AlphaFold3″ OR “foundation model”) AND (“BMP” OR “bone morphogenetic protein”).

Titles and abstracts were screened independently by two reviewers; disagreements were resolved by a third reviewer. Full texts were then assessed against the following criteria. Inclusion: English-language studies (2020–2026) that apply a computational or AI method (i) to BMP4 itself; (ii) to BMP receptors or BMP-pathway components directly relevant to BMP4 signaling; or (iii) to other targets with methods demonstrably transferable to BMP4 and discussed explicitly as such. Exclusion: non-computational descriptive studies without an AI/ML component; studies of other BMP/TGF-β ligands with no direct bearing on BMP4; opinion pieces without methodological content. To distinguish BMP4-specific findings from the broader BMP literature, each retained study was assigned one of three labels, used consistently throughout this review and in the tables (A) BMP4-specific—BMP4 itself is the subject or the molecular target (B) BMP receptor/BMP-pathway—the study targets BMP4-associated receptors or downstream BMP–SMAD signaling shared across ligands (C) Transferable AI methodology—methods developed on other targets, discussed here only as potential future applications to BMP4 and explicitly labeled “not yet applied to BMP4.” bioRxiv and arXiv preprints were included where relevant and are explicitly labeled as non-peer-reviewed at first citation. Backward and forward searches were performed on key included studies to identify additional relevant work.

Box 1 **Glossary - AI and computational terms used in this review**

**Table:** 

Graph neural network (GNN)	a neural network that operates directly on graph-structured data, learning representations of nodes and edges
Transformer	an attention-based neural architecture that processes sequences in parallel and underlies modern large language models and many protein/structure models
Random forest	an ensemble of decision trees that aggregates many weak learners; robust on tabular data and small training sets
XGBoost	a gradient-boosted tree ensemble, widely used for tabular prediction
LASSO-Cox regression	a penalized Cox proportional-hazards model that performs variable selection and shrinkage for survival analysis
DeepSurv	a deep-learning Cox model for survival prediction
RNA velocity	inference of the direction and rate of transcriptional change in single cells by distinguishing spliced from unspliced mRNA.
Single-cell foundation model	a large transformer pre-trained on millions of single-cell transcriptomes, fine-tuned for downstream tasks
Virtual screening	computational ranking of large chemical libraries against a target, either ligand-based (similarity to known actives) or structure-based (docking into a target site)
Molecular docking	prediction of a ligand’s preferred orientation and binding pose within a target binding site, with an associated score
Molecular dynamics simulation	numerical integration of Newton’s equations of motion to simulate atomic-level dynamics over time
Diffusion model	a generative model that learns to reverse a noising process to produce new samples (molecules, protein backbones, or structures)
Neuro-symbolic AI	hybrid methods that combine neural learning with symbolic/logical reasoning (e.g., logic tensor networks)

## 3 AI-driven drug discovery targeting BMP4 pathways

### 3.1 The therapeutic rationale for targeting BMP4 signaling

The BMP4 signaling axis presents a compelling but challenging target for therapeutic intervention. In oncology, BMP4 overexpression in the tumor microenvironment—particularly in cancer-associated fibroblasts (CAFs) — promotes invasion, metastasis, and immune evasion through both autocrine and paracrine mechanisms (Deng et al., 2023). In FOP, the gain-of-function ACVR1-R206H mutation renders the BMP type I receptor hypersensitive to Activin A, driving catastrophic heterotopic ossification through constitutive SMAD1/5/8 signaling (Kaplan et al., 2025). Conversely, in organ fibrosis—particularly idiopathic pulmonary fibrosis (IPF) — BMP4 exerts protective, anti-fibrotic effects by counter-regulating TGF-β1 signaling, suggesting that BMP4 agonism, rather than antagonism, may be therapeutically desirable in this context (Guan et al., 2022). This context-dependent duality—BMP4 as oncogenic driver in some tissues, tumor suppressor in others, and anti-fibrotic factor in yet another—underscores the need for tissue-specific, mechanistically precise therapeutic strategies (Figure 2).

**FIGURE 2:**
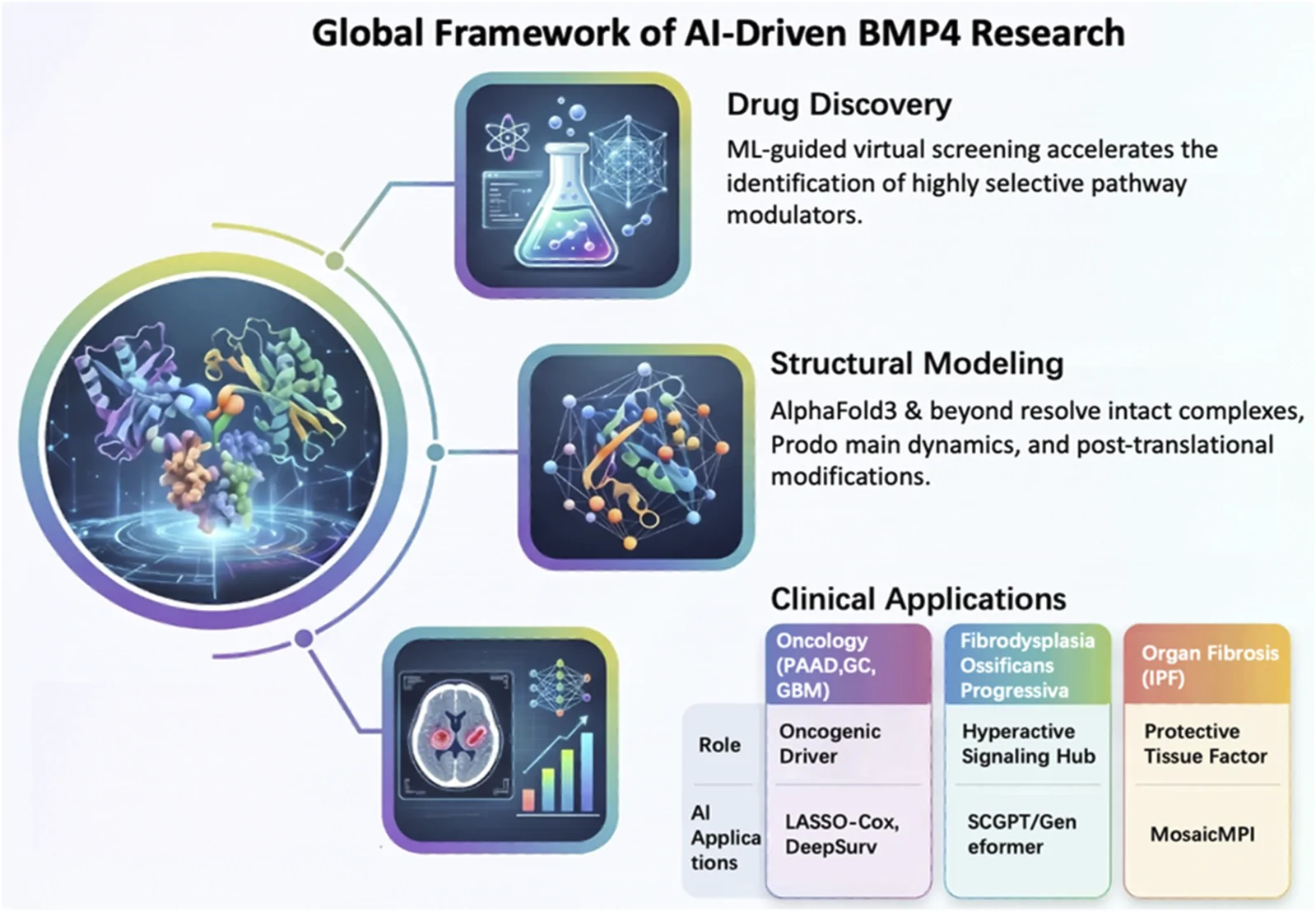
Context-dependent duality of BMP4 signaling across disease states. BMP4 acts as an oncogenic driver in cancer, a protective anti-fibrotic factor in pulmonary fibrosis, and a pathological mediator of heterotopic ossification in FOP, underscoring the need for tissue-specific, AI-guided therapeutic strategies.

### 3.2 Computational screening and ligand discovery

The application of AI to BMP4-targeted drug discovery remains in its early stages relative to more intensively studied targets such as kinases or G-protein-coupled receptors. To make clear what has actually been demonstrated for BMP4 versus what remains a proposed future application, we organize this section into three categories (A) BMP4-specific computational studies (B) BMP receptor/BMP-pathway-targeted studies, and (C) AI methods developed for other targets that are transferable to BMP4.

#### 3.2.1 BMP4-specific computational studies

The BMP4-specific computational literature is small. A 2023 study employing integrated data mining, molecular docking, and molecular dynamics (MD) simulations screened STITCH and PubChem databases to identify Retinoic acid and Torularhodin as potential BMP4 activator drugs for multi-tissue regeneration applications (Tyagi et al., 2023). It is important to note that this study used classical computational-chemistry methods—gene expression profiling (GSE34747), structure-based virtual screening, docking, and 100-ns MD—rather than deep learning or other AI methods. Nevertheless, it represents one of the first systematic computational screens specifically targeting BMP4 agonism, and it illustrates the general workflow of structure-based virtual screening: target preparation → compound library preparation → molecular docking (scoring ligand–receptor complementarity) → re-scoring and ranking → MD refinement of top poses. No BMP4-specific small-molecule modulator has yet been identified by AI-driven screening or advanced to clinical development.

#### 3.2.2 BMP receptor/BMP-pathway-targeted studies

The BMP type I receptor kinase has emerged as a tractable target for structure-based drug design in FOP. (Yang et al., 2025) reported the discovery of RK783, an orally bioavailable, selective BMP type I receptor kinase inhibitor, through a computational screening campaign of approximately 140,000 compounds at the University of Tokyo chemical library. Following the initial *in silico* screen, 1,266 compounds were synthesized and tested in SMAD1 phosphorylation assays. RK783 interacts with Y219 in the P-loop, E248 in the αC helix, and H286 in the hinge region of ACVR1, and was reported to selectively suppress BMP–SMAD1/5/9 signaling without affecting SMAD2/3. As with the Tyagi study, this campaign used structure-based *in silico* screening and biochemical validation rather than deep learning, and it exemplifies the power of integrating computational pre-screening with rigorous biochemical validation.

#### 3.2.3 AI methods developed for other targets, transferable to BMP4

In contrast to the docking-based studies above, several genuinely AI-based methods have been developed on other targets and are directly transferable to BMP4; none of these has yet been applied to BMP4 itself, and they are presented here as future opportunities rather than established BMP4 results.

The BIOPTIC B1 platform demonstrated ultra-high-throughput ligand-based virtual screening of 40 billion molecules against LRRK2, yielding hits with 110 nM affinity (Vinogradov et al., 2025). This paradigm—screening massive virtual libraries in minutes rather than months—is readily applicable to BMP4 pathway targets, provided that adequate training data can be assembled. A critical challenge for BMP4-targeted AI drug discovery is data scarcity. In a landmark study on ULK1 kinase, Naive Bayes classifiers with ECFP6 fingerprints outperformed deep learning models when only approximately 200 active compounds were available for training (Kong et al., 2024). This finding has direct relevance to BMP4, where the number of known small-molecule modulators is similarly limited. ML approaches that perform well with small training sets—random forests, support vector machines, and Bayesian classifiers—may be more immediately productive than data-hungry deep learning architectures.

At the Frontier, several emerging paradigms warrant attention: (1) neuro-symbolic AI approaches combining logic tensor networks with large language model (LLM) embeddings have achieved 97.25% accuracy in DPP-4 inhibitor classification (Hossain et al., 2025); (2) diffusion models for 3D structure-based drug design can generate novel, synthesizable molecules conditioned on target pocket geometry (Morehead and Cheng, 2024); and (3) GPT-4o-based *de novo* lead generation, when guided by inductive logic programming specifications, has produced structurally diverse, synthesizable leads for JAK2, DRD2, and DBH (Srinivasan et al., 2026). The application of these advanced methodologies to BMP4 pathway targets represents a high-priority research opportunity that remains unrealized.

### 3.3 The FOP therapeutic pipeline: a case study in computational target engagement

FOP provides the most advanced example of computationally informed drug development in the BMP signaling field. The therapeutic pipeline now includes: fidrisertib (IPN60130, Ipsen), a selective ALK2 inhibitor whose Phase II trial failed to meet its primary efficacy endpoint in late 2025; garetosmab (REGN2477, Regeneron), an anti-Activin A monoclonal antibody in Phase III; and palovarotene (Sohonos™), a retinoic acid receptor gamma agonist that received FDA approval for FOP. The discovery of ACVR1’s neomorphic response to Activin A—now recognized as the key pathological ligand in FOP—was itself facilitated by computational structural modeling of the mutant receptor that rationalizes its altered ligand specificity (Gipson et al., 2020). Additionally, a 2024 study demonstrated that iMSC-mediated delivery of ACVR2B-Fc fusion protein, a decoy receptor neutralizing multiple BMP ligands including BMP4 and BMP9, attenuates heterotopic ossification in FOP mouse models (Gao et al., 2024).

Recent mechanistic work has uncovered autophagy dysregulation as a new druggable vulnerability: the ACVR1-R206H mutant impairs autophagic flux, leading to receptor stabilization and sustained signaling; rapamycin and spermidine promote ALK2-R206H degradation via lysosomal pathways (Coculo et al., 2025). Computational modeling of these autophagy-BMP signaling feedback loops—integrating proteostasis network models with signaling dynamics—represents an underexplored opportunity for AI-driven target discovery.

## 4 Protein structure prediction and computational modeling of BMP4

### 4.1 The AlphaFold revolution and BMP4 structural biology

The release of AlphaFold2 in 2021 and the subsequent population of the AlphaFold Protein Structure Database with over 214 million predicted structures transformed structural biology overnight (Jumper et al., 2021; Varadi et al., 2022). For BMP4, a cysteine-knot growth factor whose mature, biologically active form is generated through proteolytic cleavage of a proprotein precursor by furin-like proteases, accurate structural models of both the prodomain and the mature ligand-receptor complex are essential for understanding signaling regulation.

The AlphaFold2-predicted structure of human BMP4 accurately recapitulates the canonical TGF-β family fold: a central cysteine-knot core from which two anti-parallel β-sheet fingers extend, presenting the type I and type II receptor binding epitopes (Bertoline et al., 2023). However, a critical limitation of the initial AF2 model was its inability to capture the role of the prodomain in regulating BMP4 folding, secretion, and latent complex formation. A 2025 study modeled BMP4 prodomain phosphorylation using AlphaFold3, predicting that phosphorylation at specific serine residues within the prodomain induces conformational changes that modulate furin cleavage accessibility (Kim et al., 2025). This finding illustrates a key advance of AlphaFold3 over its predecessor: the diffusion-based architecture of AF3, which replaces AF2’s structure module, enables simultaneous prediction of protein complexes with post-translational modifications, ligands, and cofactors (Abramson et al., 2024).

### 4.2 Modeling BMP4-receptor complex architecture

BMP4 signals through heterotetrameric complexes comprising two type I and two type II receptor subunits. The structural basis of BMP4’s preferential binding to BMPR1A (ALK3) over BMPR1B (ALK6) — and its interaction with BMPR2 — has been a long-standing question in the field. A 2024 structural study experimentally elucidated the mechanism by which Twisted Gastrulation 1 (TWSG1), a secreted BMP antagonist, inhibits BMP signaling: TWSG1 binds directly to the GDF5-BMPR1A interface, blocking type I receptor recruitment while permitting type II receptor engagement (Malinauskas et al., 2024). Importantly, this structural model was refined using X-ray crystallography—an experimental technique—rather than AI prediction. It nonetheless provides a template for structure-based design of BMP4 pathway modulators—whether antagonists mimicking TWSG1’s binding mode or agonists that resist TWSG1-mediated inhibition.

The broader landscape of computational tools now available for BMP4 structural biology extends well beyond AlphaFold. RoseTTAFold-All-Atom (Krishna et al., 2024) extends the RoseTTAFold architecture to non-protein molecules, enabling prediction of BMP4 in complex with small-molecule modulators or glycosaminoglycan co-receptors. ESMFold (Lin et al., 2023), which uses a protein language model to bypass multiple sequence alignment (MSA) requirements, achieves millisecond-speed predictions and may be particularly valuable for BMP4 orthologs or engineered variants with shallow MSA depth. Open-source alternatives to AF3 — Chai-1 and Boltz-1 — now enable unrestricted prediction of biomolecular complexes, democratizing access to BMP4-receptor-ligand structural modeling. These tools have not yet been systematically applied to BMP4-specific structural questions, and their utility for BMP4 remains prospective.

### 4.3 Molecular dynamics and enhanced sampling: capturing BMP4 dynamics

A fundamental limitation of all current deep learning-based structure prediction tools is their inability to capture conformational dynamics: they predict the most probable static structure, not the conformational ensemble that underpins function (Rennie and Oliver, 2025). For BMP4, whose signaling output depends on conformational changes associated with receptor binding, prodomain displacement, and antagonist engagement, this is a significant constraint.

The standard workaround—integrating AlphaFold predictions with all-atom molecular dynamics (MD) simulations—has been applied to BMP4 in several contexts. The BMP4 activator drug study described above (Section 3.2.1) employed 100-ns MD simulations to validate docking poses of Retinoic acid and Torularhodin bound to BMP4. More sophisticated enhanced sampling methods, including metadynamics, replica-exchange MD, and Gaussian accelerated MD, have been applied to related TGF-β family members and could productively be extended to BMP4. Machine learning-enhanced MD—in which neural network potentials learned from *ab initio* calculations replace classical force fields—represents a particularly promising direction for achieving quantum-chemical accuracy at MD timescales relevant to BMP4 conformational dynamics.

### 4.4 Systems-level modeling of BMP signaling dynamics

At the systems level, a landmark 2024 study in npj Systems Biology and Applications applied multi-objective Pareto optimization to the BMP/SMAD signaling network. By systematically varying non-conserved parameters—phosphatase concentration, SMAD1 and SMAD4 levels, and nuclear import factor—across 10^4^ parameter sets, the authors demonstrated that the BMP pathway cannot simultaneously optimize response speed, noise filtering, and input sensitivity. Instead, approximately 70% of parameter combinations lie on the Pareto front, meaning the pathway is inherently near-optimal across a wide range of conditions through differential expression of just a few protein components (Shaikh et al., 2024). This work elegantly explains how the virtually identical BMP pathway topology can produce system-level behaviors ranging from the rapid, noise-tolerant signaling required in *Drosophila* embryogenesis (∼30 min) to the slow, noise-filtered responses characteristic of mammalian stem cell differentiation (hours to days). From an AI perspective, this study exemplifies the power of computational optimization frameworks—borrowed from economics and machine learning—to illuminate biological design principles.

### 4.5 Limitations and validation status of AI-based structure prediction for BMP4

Given the prominence of AI structural tools in this review, it is important to state precisely what these tools do and do not establish for BMP4. First, AlphaFold-type models are predictive, not experimental: a high-confidence predicted structure is a hypothesis about a static, most-probable conformation, and does not by itself constitute an experimentally determined mechanism. Confidence is reported per-residue (pLDDT) and per-residue-pair (PAE); high global confidence does not guarantee accuracy for flexible loops, disordered regions, or post-translational states. Second, AF3’s treatment of post-translational modifications, ligands, and cofactors is predictive and requires independent validation; in the one BMP4-specific example discussed above, the AF3 prodomain-phosphorylation model was paired with experimental mutagenesis (Kim et al., 2025), but this is the exception rather than the rule. Third, the experimentally determined BMP4-relevant structures—most notably the TWSG1–BMP4–BMPR1A complex—were obtained by cryo-EM (Malinauskas et al., 2024), not by AI. Fourth, these tools do not capture conformational dynamics or the environment dependence of BMP4 signaling, which require MD or experimental measurement. In summary, AI structural prediction is best understood as a hypothesis-generation engine that accelerates—but does not replace—experimental structural biology for BMP4.

### 4.6 De novo protein design for BMP4 pathway engineering

Perhaps the most exciting, yet least exploited, frontier at the intersection of BMP4 biology and AI is *de novo* protein design. Tools such as RFdiffusion (Watson et al., 2023) and ProteinMPNN (Dauparas et al., 2022) have demonstrated remarkable capability in designing novel proteins with predetermined structures and functions—including binders, catalysts, and self-assembling nanomaterials. However, to date, no published study has applied these tools to design BMP4-based therapeutics—a statement that is restricted to the BMP4-specific category and is supported by the search strategy described in Section 2. Candidate applications include: (1) hyper-stable BMP4 variants with extended half-life for regenerative medicine applications; (2) BMP4 receptor antagonists with engineered binding specificity for ACVR1-R206H over wild-type; and (3) conditionally active BMP4 prodrugs cleavable by tumor-specific proteases. The availability of high-quality BMP4-receptor structural templates, the well-characterized BMP4 structure-function relationship, and the clinical importance of BMP4 pathway modulation together make this a high-impact, low-barrier opportunity for the protein design community.

## 5 Multi-omics and systems biology approaches to BMP4 signaling

### 5.1 Single-cell transcriptomics meets machine learning: BMP4 in the tumor microenvironment

The advent of single-cell RNA sequencing (scRNA-seq) has revolutionized our understanding of BMP4’s cell-type-specific functions in complex tissues. In scRNA-seq, individual cells are captured and barcoded, and their transcripts are reverse-transcribed and tagged with unique molecular identifiers (UMIs), yielding a high-dimensional, sparse count matrix with many zeros arising from biological absence and technical dropout. Machine learning is particularly useful for such data precisely because of this high dimensionality, sparsity, and noise: unsupervised methods identify cell types and states; trajectory-inference methods reconstruct developmental or transitional paths; and supervised classifiers or foundation models map cell states to phenotypes or perturbations.

In pancreatic adenocarcinoma (PAAD), Wang et al. (Wang et al., 2025) performed integrated single-cell and bulk RNA-seq analyses demonstrating that BMP4 is enriched in cancer cells and cancer stem cells, where it predominantly regulates metabolic pathways rather than canonical BMP-SMAD signaling. This finding—that BMP4’s prognostic significance in PAAD may derive from its metabolic functions—challenges the assumption that BMP4’s oncogenic role is mediated solely through SMAD-dependent transcription (Figure 3).

**FIGURE 3:**
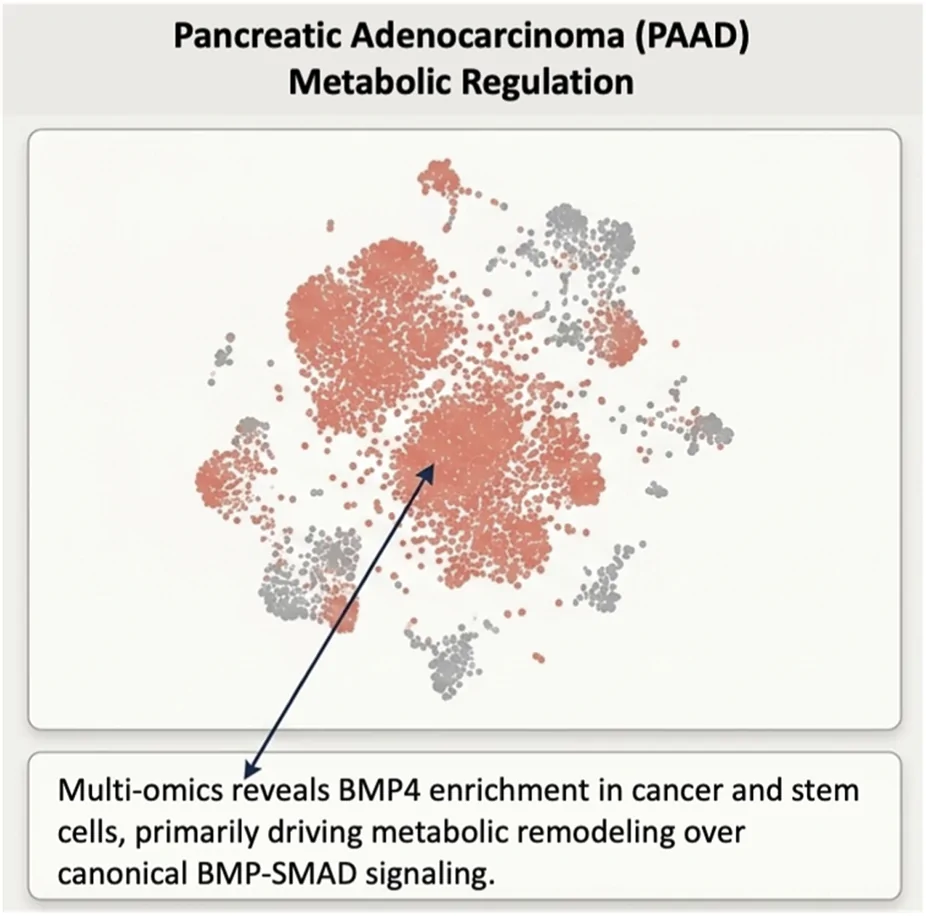
Single-cell and bulk RNA-seq analyses reveal that BMP4 is enriched in pancreatic adenocarcinoma (PAAD) cancer cells and cancer stem cells, where it predominantly regulates metabolic pathways rather than canonical BMP-SMAD signaling.

In glioblastoma (GBM), scRNA-seq studies have revealed BMP4’s exquisitely context-dependent effects on cell state transitions (Figure 4). Verploegh et al. (Verploegh et al.) profiled 17 patient-derived GBM cultures treated with BMP4 using RNA-velocity trajectory analysis, finding that: (a) in BMP4-sensitive cultures, treatment depleted pro-inflammatory and neuronal subpopulations while inducing a quiescent UA1 cluster with upregulated BMP/Hippo pathway activity; (b) OLIG1/2 expression emerged as the strongest predictor of BMP4 sensitivity; and (c) ribosomal translation genes (RPL17A, RPS27) served as early post-treatment response markers. Complementing this descriptive work, (Larsson et al., 2021) developed the STAG (State Transition and Growth) mathematical model, fitting switching rates between GBM cell states from barcoded lineage-tracing data. Their model demonstrated that BMP4 shifts the equilibrium between stem-like and invasive-like states and accurately predicted long-term population dynamics—a rare example of a mechanistic mathematical model achieving quantitative predictive power in cancer biology. Cross-species comparative analyses have likewise begun to reveal conserved therapeutic vulnerabilities in GBM (Foerster et al., 2025).

**FIGURE 4:**
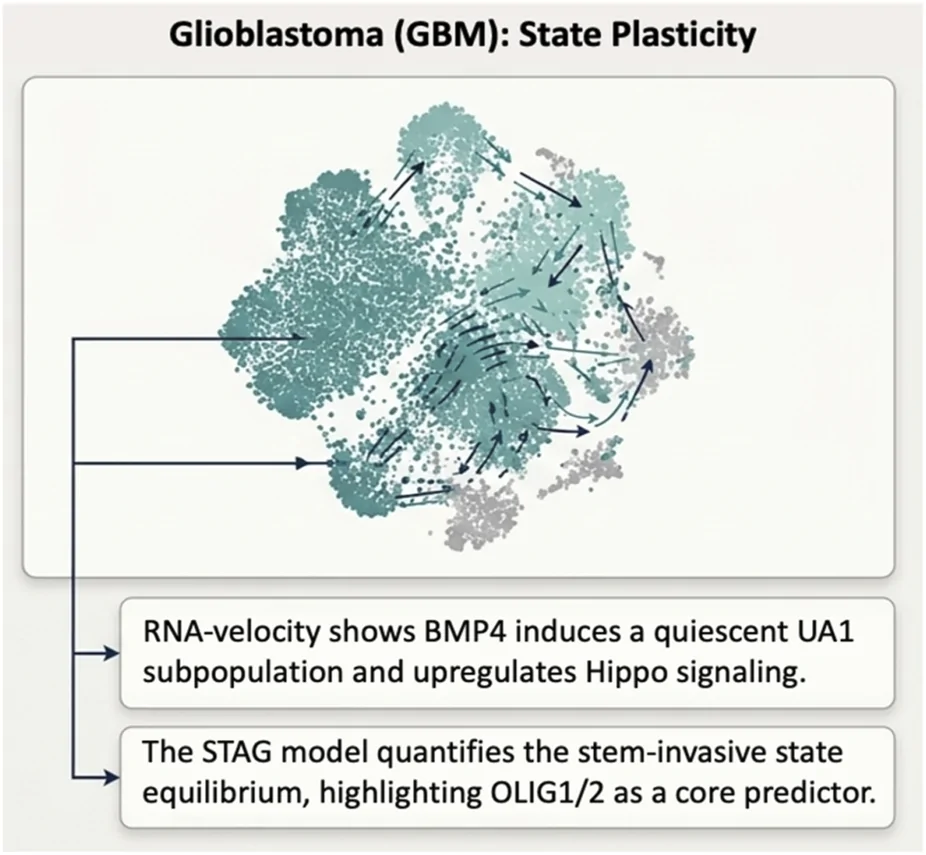
RNA-velocity trajectory analysis of BMP4-treated patient-derived glioblastoma (GBM) cultures reveals context-dependent cell state transitions, including depletion of pro-inflammatory and neuronal subpopulations and induction of a quiescent, BMP/Hippo pathway-active cluster in BMP4-sensitive cultures.

Most recently, a 2025 multi-omics study integrated snRNA-seq (Neftel 4-state classification), bulk RNA-seq deconvolution (Bisque), phosphoproteomics, and Boolean network modeling (MaBoSS) to predict GBM cell state transitions under pharmacological perturbation (Bumbaca et al., 2025). The integrated Boolean model enabled *in silico* knockout simulations, proposing interventions that drive or block specific state transitions—a framework for cell-state-directed therapy that could be generalized to other BMP4-associated cancers.

### 5.2 Hypoxia, immune evasion, and BMP4 in gastric cancer

In gastric cancer (GC), Deng et al. (2023) combined scRNA-seq and bulk RNA-seq with LASSO-COX regression to construct a hypoxia-immune-related prognostic panel incorporating BMP4 alongside POSTN, MXRA5, LBH, and APOD (Deng et al., 2023). Their analysis revealed that BMP4 is significantly upregulated in GC-associated CAFs compared to normal fibroblasts, and that high hypoxia scores correlated with decreased anti-tumor immune infiltration (activated NK cells, CD8^+^ T cells) and elevated M2 macrophage polarization. Critically, patients with high hypoxia scores exhibited higher Tumor Immune Dysfunction and Exclusion (TIDE) scores, predicting poorer responses to immune checkpoint blockade—a finding with direct clinical implications for immunotherapy patient stratification, although it derives from retrospective cohorts and has not been prospectively validated. This study exemplifies the power of integrating multi-omics data with machine learning to discover clinically actionable gene signatures embedded within the tumor microenvironment.

### 5.3 Transcriptomic signatures of BMP4 in fibrotic disease

In idiopathic pulmonary fibrosis (IPF), BMP4 has emerged as a consistently dysregulated, protective factor. A 2023 integrated bioinformatics analysis across multiple GEO datasets identified BMP4 among 18 hub genes in the IPF protein-protein interaction network, validated by qPCR in TGF-β1-induced lung fibroblasts and a bleomycin mouse model (Zhang et al., 2023). (Ghandikota et al., 2022) applied weighted gene co-expression network analysis (WGCNA) to two independent IPF transcriptomic cohorts, finding that BMP signaling was significantly enriched in the disease-associated co-expression module (FDR: 2.74 × 10^−2^). In a bleomycin-induced IPF mouse model, Bmp4 was significantly downregulated—and not restored by iPSC therapy—suggesting that BMP4 suppression may be a persistent, therapeutically relevant feature of fibrotic remodeling (Bayati et al., 2023).

Functionally, (Guan et al., 2022) reported that BMP4 inhibits pulmonary fibrosis through dual modulation of cellular senescence and mitophagy in lung fibroblasts. This mechanistic finding positions BMP4 agonism as a potential therapeutic strategy for IPF—a notable contrast to the BMP4 antagonism sought in FOP. The integration of these transcriptomic and functional datasets using AI-driven multi-omics integration frameworks—such as MosaicMPI (Verhey et al., 2024), which deconvolves multi-omics signals through matrix factorization—could systematically map BMP4’s context-dependent regulatory logic across tissues and disease states.

### 5.4 Single-cell foundation models: the next frontier for BMP4 biology

The emergence of single-cell foundation models—large transformer architectures pre-trained on tens of millions of single-cell transcriptomes—represents a paradigm shift in computational biology. Geneformer (Theodoris et al., 2023), scGPT (Cui et al., 2024), and UCE (Rosen et al., 2023) learn context-aware gene and cell representations that can be fine-tuned for downstream tasks including gene perturbation prediction, cell type annotation, and disease state classification. In principle, these models could predict BMP4 perturbation effects across cell types, tissues, and disease contexts *in silico*, dramatically accelerating hypothesis generation. However, none has yet been applied to BMP4-specific questions.

Moreover, recent benchmarks have introduced important caveats. A 2025 systematic evaluation in Nature Methods demonstrated that deep learning-based gene perturbation effect prediction does not yet outperform simple linear baselines on standard benchmarks (Ahlmann-Eltze et al., 2025). A companion study in Nature Communications found that single-cell foundation model attention mechanisms primarily capture co-expression patterns rather than unique regulatory signals (Wang et al., 2026). These findings suggest that, while foundation models hold immense promise for BMP4 biology—particularly for predicting context-dependent BMP4 signaling responses—their current implementations should be interpreted cautiously, and predictions should be validated experimentally wherever feasible (Table 2).

**TABLE 2:** Representative AI methods, application domains, and BMP4-specific status.

Method	Application domain	BMP4 context	BMP4-specific status	Representative citation
AlphaFold3 (diffusion)	Structure prediction	BMP4/BMP4-receptor/prodomain	Predicted; partially validated	(Abramson et al., 2024); (Kim et al., 2025)
RFdiffusion + ProteinMPNN	*De novo* design	Not yet applied	Unexplored	(Watson et al., 2023); (Dauparas et al., 2022)
LASSO-Cox regression	Prognostic modeling	Pancreatic, gastric cancer nomograms	BMP4 in panel; retrospective	(Wang et al., 2025); (Deng et al., 2023)
DeepSurv (DL survival)	Survival prediction	PDAC risk stratification	Disease-associated; retrospective	(Xiao et al., 2025)
Random Forest/XGBoost	Diagnostic classification	PDAC diagnosis (AUC 0.889)	Disease-associated	(Xiao et al., 2025)
RNA velocity (scVelo)	Cell-state trajectories	GBM BMP4 response	BMP4-specific	(Verploegh et al., 2022)
STAG model (ODE-based)	Dynamic cell-state modeling	GBM state transitions	BMP4-modulated	(Larsson et al., 2021)
Boolean networks (MaBoSS)	Signaling network simulation	GBM cell-state-directed therapy	Disease-associated	(Bumbaca et al., 2025)
WGCNA	Co-expression analysis	IPF disease modules	BMP-pathway	(Ghandikota et al., 2022)
Pareto optimization	Signaling pathway analysis	BMP–SMAD performance trade-offs	BMP-pathway	(Shaikh et al., 2024)
Geneformer/scGPT	Single-cell foundation model	BMP4 perturbation prediction (in principle)	Not yet applied	(Theodoris et al., 2023); (Cui et al., 2024)
BIOPTIC B1 (transformer)	Ultra-large virtual screening	40B-molecule LRRK2 screen	Transferable	(Vinogradov et al., 2025)

Abbreviations: AUC, area under the receiver operating characteristic curve; DL, deep learning; GBM, glioblastoma multiforme; IPF, idiopathic pulmonary fibrosis; LASSO, least absolute shrinkage and selection operator; MaBoSS, markovian boolean stochastic simulator; MD, molecular dynamics; ODE, ordinary differential equation; PDAC, pancreatic ductal adenocarcinoma; sc = single-cell; STAG, state transition and growth; WGCNA, weighted gene co-expression network analysis.

## 6 Clinical AI applications for BMP4-Associated diseases

### 6.1 Pancreatic adenocarcinoma: BMP4 as a prognostic biomarker

Pancreatic ductal adenocarcinoma (PDAC) remains one of the deadliest malignancies, with a 5-year survival rate below 12%. In this context, the identification of robust prognostic biomarkers is of paramount importance. Wang et al. (2025) performed the most comprehensive analysis to date of BMP4’s prognostic role in PAAD, integrating TCGA-PAAD, GSE57495, and GSE78229 cohorts (Wang et al., 2025). Their key findings include: (a) BMP4 is minimally expressed in normal pancreas but markedly upregulated in PAAD; (b) high BMP4 expression independently predicts poor overall survival, disease-free survival, and progression-free survival; and (c) a nomogram incorporating BMP4 with clinical variables (sex, TNM stage, age) achieved C-index values of 0.738 (training) and 0.724 (validation), indicating promising, though as-yet externally unvalidated, predictive accuracy.

Complementing this biomarker work, Xiao et al. (2025) compared multiple machine learning models for PDAC diagnosis and survival prediction (Xiao et al., 2025). Random Forest achieved the highest diagnostic performance (AUC: 0.889; accuracy: 84.21%), while DeepSurv—a deep learning-based Cox proportional hazards model—outperformed traditional Cox regression for survival prediction (C-index: 0.738 vs. lower Cox values). DeepSurv was further used to generate personalized treatment recommendations based on predicted risk scores. The integration of BMP4 expression into such deep learning survival frameworks—which Wang et al. did not explore—could further enhance predictive performance, pending prospective validation.

### 6.2 Glioblastoma: AI-driven cell state classification and therapeutic prediction

Glioblastoma is characterized by remarkable cellular plasticity, with individual tumors harboring multiple coexisting cell states that interconvert dynamically. BMP4 has emerged as a key regulator of this plasticity, capable of inducing astroglial differentiation, quiescence, and p21-dependent senescence depending on cellular context. The integration of machine learning with single-cell and multi-omics profiling has begun to unravel this complexity.

The STAG mathematical model (Larsson et al., 2021) demonstrated that GBM cell states follow a hierarchical yet plastic organization, with patient-specific transition rates that are modulated by BMP4. The 2025 multi-omics Boolean network study extended this framework by building cell-state-specific protein-protein interaction networks from phosphoproteomics data, converting them to executable Boolean models, and training machine learning classifiers to predict dominant cell states from independent clinical datasets (Foerster et al., 2025). This pipeline—snRNA-seq → deconvolution → phosphoproteomics → Boolean network → ML classifier—represents a generalizable template for computationally driven, cell-state-directed therapy in cancer.

The discovery that BMP4 induces a p21-dependent senescence-like phenotype specifically in therapy-resistant mesenchymal-like (MES) GBM cells, and that senolytic treatment with navitoclax selectively eliminates these cells (Niklasson et al., 2025), opens a therapeutic window that could be optimized using AI. For example, machine learning models trained on pre-treatment scRNA-seq profiles could predict which patients are most likely to benefit from BMP4-senolytic combination therapy—a form of computational precision oncology that remains unexplored.

### 6.3 Breast cancer: BMP4-SMAD4 axis and prognostic modeling

In breast cancer, BMP4 exhibits a complex, subtype-dependent role that has only begun to be interrogated with AI methods. A 2024 study demonstrated that loss of tumor-derived SMAD4 enhances primary tumor growth but not metastasis following BMP4 signaling, uncoupling BMP4’s proliferative and invasive effects in a SMAD4-dependent manner (Chi et al., 2024). This mechanistic nuance has important implications for prognostic modeling: if BMP4’s prognostic value depends on SMAD4 status, single-gene biomarkers may misclassify SMAD4-deficient tumors.

From a machine learning perspective, large-scale breast cancer transcriptomic studies using the METABRIC and TCGA-BRCA cohorts have incorporated BMP4 into broader prognostic frameworks (Shah et al., 2026). Ensemble classifiers—including XGBoost, random forest, and LASSO—applied to immune-related gene panels that include BMP4 have achieved AUC values of 0.75–0.85 for survival prediction (Hosni et al., 2019). However, unlike in pancreatic cancer, where BMP4 has been evaluated as a standalone nomogram variable, its independent contribution to breast cancer prognostic models has not been systematically isolated. Integrating BMP4 expression with SMAD4 mutational status, hormone receptor subtype, and tumor microenvironment features within multi-task learning architectures could substantially refine risk stratification.

### 6.4 Gastric cancer: hypoxia-immune prognostic signatures

As described in Section 4.2, the Deng et al. (Deng et al., 2023) study in gastric cancer is notable for its translational orientation. The BMP4-containing 5-gene hypoxia panel not only stratifies patients by survival but also predicts immune infiltration patterns and immunotherapy/chemotherapy response. The integration of TIDE scores—a computational method for predicting immunotherapy response from bulk tumor transcriptomes—with the hypoxia-immune prognostic panel creates a dual-purpose biomarker: prognostic and predictive. These results are retrospective and require prospective, independent validation before clinical use. Extensions of this work could incorporate deep learning-based pathology image analysis, in which whole-slide images (WSIs) are analyzed by convolutional neural networks to directly predict BMP4-related hypoxia signatures from H&E-stained sections—bypassing the need for sequencing-based biomarker assessment.

### 6.5 Organ fibrosis: BMP4 as a protective factor and therapeutic target

As reviewed in Section 5.3, BMP4’s protective role in pulmonary fibrosis positions it as a potential therapeutic target, but the clinical translation of this biology has been limited. In IPF, two FDA-approved anti-fibrotic drugs—pirfenidone and nintedanib—slow disease progression but do not halt or reverse fibrosis. BMP4 agonism represents a mechanistically orthogonal approach: rather than directly inhibiting pro-fibrotic pathways (as nintedanib inhibits multiple tyrosine kinases), BMP4 agonism would reinforce an endogenous anti-fibrotic program.

AI could accelerate this translation in several ways: (1) virtual screening for small-molecule BMP4 agonists suitable for inhaled delivery; (2) machine learning analysis of IPF patient lung transcriptomes to identify subgroups most likely to benefit from BMP4 pathway augmentation; and (3) deep learning-based analysis of serial CT imaging to track BMP4 agonist treatment response with greater sensitivity than conventional pulmonary function tests. The pan-organ transcriptomic atlas of allograft dysfunction published in Nature Medicine (2024), which decoded shared and organ-specific hallmarks of fibrosis across kidney, liver, lung, and heart transplants, provides a multi-tissue framework for understanding BMP4’s context-dependent roles in fibrosis (Robertson et al., 2024).

### 6.6 Fibrodysplasia ossificans progressiva: computational target validation

FOP, though an ultra-rare disease (prevalence ∼1 in 2 million), has attracted intensive drug development effort because of its devastating phenotype and well-defined genetic cause. The computational and AI contributions to FOP therapeutic development include: (a) ligand-based virtual screening of 140,000 compounds that led to RK783 (Yang et al., 2025); (b) structural models elucidating how the ACVR1-R206H mutation creates a neomorphic response to Activin A (Gipson et al., 2020); (c) mTORC1-autophagy feedback loop modeling identifying rapamycin and spermidine as ALK2-degrading agents (Meng et al., 2022); and (d) the growing recognition that OXPHOS upregulation in FOP-MSCs creates a metabolic vulnerability targetable by IACS-010759 (Sun et al., 2024).

These advances illustrate a broader principle: AI is most effective in rare disease drug development when integrated with deep mechanistic understanding. For BMP4-related rare diseases, the marriage of AI-driven screening with hypothesis-driven biological investigation offers the most promising path to clinical impact.

Beyond the ACVR1-focused approaches described above, BMP4 itself—as one of the osteogenic ligands whose signaling is amplified by the mutant receptor—remains a relevant node for therapeutic intervention. Computational modeling of the full BMP ligand-receptor interaction network, incorporating BMP4, BMP2, BMP7, BMP9, Activin A, and their respective type I/type II receptor preferences, could identify dominant signaling hubs and predict which ligand-level interventions would most effectively suppress pathological heterotopic ossification while preserving homeostatic BMP signaling. Such multi-ligand, multi-receptor network models have not yet been built for the FOP context but are technically feasible using existing structural and biochemical data.

## 7 Emerging frontiers and future directions

### 7.1 De novo design of bmp4-based therapeutics

The application of RFdiffusion and ProteinMPNN to design BMP4-derived therapeutics represents the most conspicuous gap in this field. RFdiffusion, a denoising diffusion probabilistic model fine-tuned on protein backbone structures, generates novel protein backbones conditioned on target binding site geometry (Watson et al., 2023). When paired with ProteinMPNN, a message-passing neural network that designs amino acid sequences folding into specified backbones, the RFdiffusion-ProteinMPNN pipeline can create binders, enzymes, and self-assembling materials with atomic-level precision (Dauparas et al., 2022). This pipeline has not yet been applied to BMP4 biology despite readily available structural templates—BMP4 in complex with BMPR1A and BMPR2, the TWSG1-BMP4 interface (Malinauskas et al., 2024), and the ACVR1-R206H mutant structure.

A focused protein design campaign targeting BMP4 could yield several classes of therapeutics. First, hyper-stable BMP4 variants with engineered disulfide bonds or helix-capping motifs could extend the short *in vivo* half-life of wild-type BMP4, addressing a major limitation for regenerative medicine applications. Second, BMP4-based decoy receptors or ligand traps with femtomolar affinity for Activin A could provide next-generation FOP therapies with reduced immunogenicity compared to monoclonal antibodies. Third, conditionally activated BMP4 prodrugs—in which receptor binding is sterically blocked by a tumor protease-cleavable prodomain—could unleash anti-proliferative BMP4 signaling specifically within the tumor microenvironment, sparing normal tissues from on-target toxicity. Fourth, BMP4 variants with biased receptor specificity could dissect the distinct biological outputs of different receptor combinations while opening therapeutic windows. The field’s current inability to design such molecules is not due to fundamental technical barriers but rather to a gap in research attention; closing this gap should be a priority.

### 7.2 Multimodal foundation models for BMP4 biology

The next-generation of biomedical AI will almost certainly be multimodal—integrating text, sequence, structure, imaging, and clinical data within unified representational frameworks. For BMP4 research, a multimodal model could simultaneously: (a) predict the structure of a novel BMP4 variant from its sequence; (b) estimate its binding affinity to each BMP receptor subtype; (c) forecast it signaling output (SMAD1/5/8 phosphorylation dynamics) in a given cell type; and (d) correlate these molecular properties with clinical imaging phenotypes from electronic health records. While such models do not yet exist, their building blocks—AlphaFold3 for structure, BindCraft for binding prediction, Geneformer for transcriptomic context, and vision transformers for pathology imaging—are individually maturing. Integrating them into a cohesive BMP4-centric foundation model represents a grand challenge for the field.

### 7.3 AI-native experimental design

A Frontier that has received minimal attention in BMP4 research is AI-native experimental design—the use of active learning, Bayesian optimization, and reinforcement learning to guide experiments. In drug discovery, Bayesian optimization has been used to navigate chemical space efficiently by iteratively selecting compounds for synthesis and testing based on predicted information gain. In structural biology, active learning has guided cryo-EM data collection to maximize map quality per unit of microscope time. Applying these frameworks to BMP4 research—for example, using active learning to efficiently sample the combinatorial space of BMP4 mutations that alter receptor specificity, or using Bayesian optimization to design minimal promoter constructs for BMP4-responsive gene circuits—could dramatically accelerate discovery at reduced experimental cost.

### 7.4 Data resources and community infrastructure

Realizing the full potential of AI-augmented BMP4 research will require community-level data infrastructure that does not currently exist. Priorities include: (a) a curated BMP4 interactome database integrating protein-protein interactions, post-translational modifications, and transcriptional targets across cell types and species; (b) standardized benchmarking datasets for BMP4 signaling prediction tasks; (c) open-source, containerized workflows for BMP4 structural modeling, MD simulation, and systems biology analysis that lower the barrier to entry for experimental BMP4 biologists; and (d) community-wide challenges analogous to the D3R Grand Challenges that have advanced computational drug design.

### 7.5 Ethical considerations and responsible AI use

The integration of AI into BMP4 biomedical research raises several ethical considerations. AI-generated structural models and predicted biomarkers require rigorous experimental validation before influencing clinical decision-making; the danger of “model hallucination” is real and must be guarded against (Lin et al., 2023). AI-discovered BMP4 modulators should be screened for off-target effects using unbiased proteomics and transcriptomics before entering clinical development. In clinical AI applications, algorithmic fairness must be ensured: prognostic models trained predominantly on datasets of European ancestry (as is the case for most TCGA-derived models) may perform poorly in underrepresented populations. Finally, AI-augmented BMP4 research should adhere to emerging guidelines for responsible AI use in biomedical science, including transparent reporting of model architectures, training data, and validation procedures.

## 8 Conclusion

The intersection of BMP4 biology and artificial intelligence stands at a pivotal moment. Deep learning has transformed protein structure prediction to the point where high-confidence predicted models of BMP4, its receptors, and their complexes are available at the click of a button—offering provisional, computationally derived answers to decades-old questions about ligand-receptor interface architecture and antagonist binding mechanisms, pending experimental confirmation. Machine learning-driven virtual screening, though not yet yielding clinical BMP4 modulators, has identified tractable chemical starting points and established computational pipelines that are readily transferable to this target class. In the clinical domain, LASSO-COX regression and deep survival models incorporating BMP4 expression have promising prognostic value in retrospective analyses of pancreatic cancer, gastric cancer, and glioblastoma, while single-cell multi-omics analyses have revealed BMP4’s context-dependent roles in tumor cell state plasticity and immune evasion.

Yet the field has only scratched the surface. No published study has applied RFdiffusion or ProteinMPNN to engineer BMP4-based therapeutics. No BMP4-specific small-molecule inhibitor has entered clinical trials. No multimodal foundation model has been trained to predict BMP4 signaling outcomes across biological contexts. No active learning framework has been deployed to optimize BMP4-focused experiments. These gaps are not failures—they are opportunities. The foundational tools are in place; the biological questions are well-posed; and a substantial, clinically significant unmet need exists across these diseases. The next 5 years (approximately 2026–2031, mirroring the 2020–2026 window reviewed here) will determine whether the convergence of BMP4 biology and AI fulfills its substantial translational promise.

## References

[B1] Abramson J. Adler J. Dunger J. Evans R. Green T. Pritzel A. (2024). Accurate structure prediction of biomolecular interactions with AlphaFold 3. Nature 630, 493–500. 10.1038/s41586-024-07487-w PMC11168924 38718835

[B2] Ahlmann-Eltze C. Huber W. Anders S. (2025). Deep-learning-based gene perturbation effect prediction does not yet outperform simple linear baselines. Nat. Methods 22, 1657–1661. 10.1038/s41592-025-02772-6 40759747 PMC12328236

[B3] Bayati P. Taherian M. Soleimani M. Farajifard H. Mojtabavi N. (2023). Induced pluripotent stem cells modulate the wnt pathway in the bleomycin-induced model of idiopathic pulmonary fibrosis. Stem Cell Res. and Ther. 14, 343. 10.1186/s13287-023-03581-4 PMC10685538 38017561

[B4] Bertoline L. M. F. Lima A. N. Krieger J. E. Teixeira S. K. (2023). Before and after AlphaFold2: an overview of protein structure prediction. Front. Bioinforma. 3, 1120370. 10.3389/fbinf.2023.1120370 36926275 PMC10011655

[B5] Bumbaca B. Huggins J. R. Birtwistle M. R. Gallo J. M. (2025). Network analyses of brain tumor multiomic data reveal pharmacological opportunities to alter cell state transitions. Npj Syst. Biol. Appl. 11, 14. 10.1038/s41540-025-00493-2 PMC11787326 39893170

[B6] Chi L. H. Redfern A. D. Roslan S. Street I. P. Burrows A. D. Anderson R. L. (2024). Loss of tumor-derived SMAD4 enhances primary tumor growth but not metastasis following BMP4 signalling. Cell Commun. Signal. 22, 248. 10.1186/s12964-024-01559-0 38689334 PMC11060976

[B7] Coculo L. Wits M. Mariani I. Fianco G. Cappato S. Bocciardi R. (2025). Interplay between ALK2R206H mutant receptor and autophagy signaling regulates receptor stability and its chondrogenic functions. Cell Death Discov. 11, 117. 10.1038/s41420-025-02393-0 40121219 PMC11929866

[B8] Cui H. Wang C. Maan H. Pang K. Luo F. Duan N. (2024). scGPT: toward building a foundation model for single-cell multi-omics using generative AI. Nat. Methods 21, 1470–1480. 10.1038/s41592-024-02201-0 38409223

[B9] Dauparas J. Anishchenko I. Bennett N. Bai H. Ragotte R. J. Milles L. F. (2022). Robust deep learning–based protein sequence design using ProteinMPNN. Science 378, 49–56. 10.1126/science.add2187 PMC9997061 36108050

[B10] Deng C. Deng G. Chu H. Chen S. Chen X. Li X. (2023). Construction of a hypoxia-immune-related prognostic panel based on integrated single-cell and bulk RNA sequencing analyses in gastric cancer. Front. Immunol. 14, 1140328. 10.3389/fimmu.2023.1140328 PMC10169567 37180146

[B11] Foerster L. C. Kaya O. Wust V. Danciu D. P. Bekavac M. Ziegler K. C. (2025). Cross-species comparison reveals therapeutic vulnerabilities halting glioblastoma progression. bioRxiv (preprint). 10.1101/2023.09.15.557713 PMC12329047 40770193

[B12] Gao P. Inada Y. Hotta A. Sakurai H. Ikeya M. (2024). iMSC-mediated delivery of ACVR2B-Fc fusion protein reduces heterotopic ossification in a mouse model of fibrodysplasia ossificans progressiva. Stem Cell Res. and Ther. 15, 83. 10.1186/s13287-024-03691-7 PMC10949803 38500216

[B13] Ghandikota S. Sharma M. Ediga H. H. Madala S. K. Jegga A. G. (2022). Consensus gene co-expression network analysis identifies novel genes associated with severity of fibrotic lung disease. Int. J. Mol. Sci. 23, 5447. 10.3390/ijms23105447 35628257 PMC9141193

[B14] Gipson G. R. Goebel E. J. Hart K. N. Kappes E. C. Kattamuri C. McCoy J. C. (2020). Structural perspective of BMP ligands and signaling. Bone 140, 115549. 10.1016/j.bone.2020.115549 32730927 PMC7502536

[B15] Guan R. Yuan L. Li J. Wang J. Li Z. Cai Z. (2022). Bone morphogenetic protein 4 inhibits pulmonary fibrosis by modulating cellular senescence and mitophagy in lung fibroblasts. Eur. Respir. J. 60, 2102307. 10.1183/13993003.02307-2021 35777761 PMC9808813

[B16] Hosni M. Abnane I. Idri A. Carrillo de Gea J. M. Fernández Alemán J. L. (2019). Reviewing ensemble classification methods in breast cancer. Comput. Methods Programs Biomed. 177, 89–112. 10.1016/j.cmpb.2019.05.019 31319964

[B17] Hossain D. Saghapour E. Chen J. Y. Y. (2025). NeSyDPP-4: discovering DPP-4 inhibitors for diabetes treatment with a neuro-symbolic AI approach. Front. Bioinforma. 5, 1603133. 10.3389/fbinf.2025.1603133 40761758 PMC12319772

[B18] Jumper J. Evans R. Pritzel A. Green T. Figurnov M. Ronneberger O. (2021). Highly accurate protein structure prediction with AlphaFold. Nature 596, 583–589. 10.1038/s41586-021-03819-2 PMC8371605 34265844

[B19] Kaplan F. S. Shore E. M. Pignolo R. J. (2025). Fibrodysplasia ossificans progressiva emerges from obscurity. Trends Mol. Med. 31, 106–116. 10.1016/j.molmed.2024.08.010 39299836

[B20] Kim H.-S. Sanchez M. L. Silva J. Schubert H. L. Dennis R. Hill C. P. (2025). Mutations that prevent phosphorylation of the BMP4 prodomain impair proteolytic maturation of homodimers leading to lethality in mice. eLife 14, RP105018. 10.7554/eLife.105018 PMC12122004 40439112

[B21] Kong M.-M. Wei T. Liu B. Xi Z. X. Ding J. T. Liu X. (2024). Discovery of novel ULK1 inhibitors through machine learning-guided virtual screening and biological evaluation. Future Med. Chem. 16, 1821–1837. 10.1080/17568919.2024.2385288 39145469 PMC11485869

[B22] KrishnaR. WangJ. AhernW. SturmfelsP. VenkateshP. KalvetI. (2024). Generalized biomolecular modeling and design with RoseTTAFold all-atom. Science 384, eadl2528. 10.1126/science.adl2528 38452047

[B23] Larsson I. Dalmo E. Elgendy R. Niklasson M. Doroszko M. Segerman A. (2021). Modeling glioblastoma heterogeneity as a dynamic network of cell states. Mol. Syst. Biol. 17, MSB202010105. 10.15252/msb.202010105 34528760 PMC8444284

[B24] Lin Z. Akin H. Rao R. Hie B. Zhu Z. Lu W. (2023). Evolutionary-scale prediction of atomic-level protein structure with a language model. Science 379, 1123–1130. 10.1126/science.ade2574 36927031

[B25] Malinauskas T. Moore G. Rudolf A. F. Eggington H. Belnoue-Davis H. L. El Omari K. (2024). Molecular mechanism of BMP signal control by twisted gastrulation. Nat. Commun. 15, 4976. 10.1038/s41467-024-49065-8 PMC11167000 38862520

[B26] Meng X. Wang H. Hao J. (2022). Recent progress in drug development for fibrodysplasia ossificans progressiva. Mol. Cell. Biochem. 477, 2327–2334. 10.1007/s11010-022-04446-9 35536530 PMC9499916

[B27] Miyazono K. Kamiya Y. Morikawa M. (2010). Bone morphogenetic protein receptors and signal transduction. J. Biochem. 147, 35–51. 10.1093/jb/mvp148 19762341

[B28] Morehead A. Cheng J. (2024). Geometry-complete diffusion for 3D molecule generation and optimization. Commun. Chem. 7, 150. 10.1038/s42004-024-01233-z PMC11222514 38961141

[B29] Niklasson M. Dalmo E. Segerman A. Rendo V. Westermark B. (2025). p21-dependent senescence induction by BMP4 renders glioblastoma cells vulnerable to senolytics. Int. J. Mol. Sci. 26, 3974. 10.3390/ijms26093974 PMC12071447 40362216

[B30] Rennie M. L. Oliver M. R. (2025). Emerging frontiers in protein structure prediction following the AlphaFold revolution. J. R. Soc. Interface 22, 20240886. 10.1098/rsif.2024.0886 PMC11999738 40233800

[B31] Robertson H. Kim H. J. Li J. Robertson N. Robertson P. Jimenez-Vera E. (2024). Decoding the hallmarks of allograft dysfunction with a comprehensive pan-organ transcriptomic atlas. Nat. Med. 30, 3748–3757. 10.1038/s41591-024-03030-6 38890530 PMC11645273

[B32] Rosen Y. Roohani Y. Agrawal A. Samotorcan L. Quake S. R. Leskovec J. (2023). Universal cell embeddings: a foundation model for cell biology. bioRxiv (preprint). 10.1101/2023.11.28.568918 PMC13441871 42420460

[B33] Shah S. Samah A. A. Madni S. H. H. Hashim S. Z. M. Faheem M. (2026). Recent advancements in artificial intelligence-driven breast cancer molecular subtypes classification using multi-omics: a comprehensive review. Eng. Appl. Artif. Intell. 170, 114237. 10.1016/j.engappai.2026.114237

[B34] Shaikh R. Larson N. J. Kam J. Hanjaya-Putra D. Zartman J. Umulis D. M. (2024). Optimal performance objectives in the highly conserved bone morphogenetic protein signaling pathway. Npj Syst. Biol. Appl. 10, 103. 10.1038/s41540-024-00430-9 39277657 PMC11401948

[B35] Srinivasan A. Dash T. Baskar A. Bain M. Dey S. K. Banerjee M. , (2026). Symbolic Neural Generation with Applications to Lead Discovery in Drug Design.

[B36] Sun L. Jin Y. Nishio M. Watanabe M. Kamakura T. Nagata S. (2024). Oxidative phosphorylation is a pivotal therapeutic target of fibrodysplasia ossificans progressiva. Life Sci. Alliance 7, e202302219. 10.26508/lsa.202302219 PMC10875110 38365425

[B37] Theodoris C. V. Xiao L. Chopra A. Chaffin M. D. Al Sayed Z. R. Hill M. C. (2023). Transfer learning enables predictions in network biology. Nature 618, 616–624. 10.1038/s41586-023-06139-9 PMC10949956 37258680

[B38] Tyagi N. Gambhir K. Sharma D. Gangenahalli G. Verma Y. K. (2023). Data mining and structural analysis for multi-tissue regeneration potential of BMP-4 and activator drugs. J. Biomol. Struct. Dyn. 41, 4405–4420. 10.1080/07391102.2022.2067899 35491689

[B39] Varadi M. Anyango S. Deshpande M. Nair S. Natassia C. Yordanova G. (2022). AlphaFold protein structure database: massively expanding the structural coverage of protein-sequence space with high-accuracy models. Nucleic Acids Res. 50, D439–D444. 10.1093/nar/gkab1061 PMC8728224 34791371

[B40] Verhey T. B. Seo H. Gillmor A. Thoppey-Manoharan V. Schriemer D. Morrissy S. (2024). mosaicMPI: a framework for modular data integration across cohorts and -omics modalities. Nucleic Acids Res. 52, e53. 10.1093/nar/gkae442 PMC11229337 38813827

[B41] Verploegh I. S. C. Conidi A. Brouwer R. W. W. Balcioglu H. E. Karras P. Makhzami S. (2022). Comparative single-cell RNA-Sequencing profiling of BMP4-treated primary glioma cultures reveals therapeutic markers. Neuro-Oncology 24, 2133–2145. 10.1093/neuonc/noac143 PMC9713526 35639831

[B42] Vinogradov V. Nguyen K. T. Steshin S. Izmailov I. Doronichev A. (2025). BIOPTIC B1 ultra-high-throughput virtual screening system discovers LRRK2 ligands in vast chemical space. J. Chem. Inf. Model. 65, 9927–9936. 10.1021/acs.jcim.5c00743 PMC12529759 40939039

[B43] Wang R. N. Green J. Wang Z. Deng Y. Qiao M. Peabody M. (2014). Bone morphogenetic protein (BMP) signaling in development and human diseases. Genes and Dis. 1, 87–105. 10.1016/j.gendis.2014.07.005 25401122 PMC4232216

[B44] Wang A. Shen X. Lu S. Bi C. Cao Y. Liu Y. (2025). Comprehensive analyses of single-cell and bulk RNA-Seq reveal the biological and prognostic roles of BMP4 in pancreatic adenocarcinoma. Front. Mol. Biosci. 12, 1686938. 10.3389/fmolb.2025.1686938 41169612 PMC12568421

[B45] Wang J. Tan C. Gao Z. Shao S. Liu S. Li S. Z. (2026). Illuminating cell states by a comprehensive and interpretable single cell foundation model. Nat. Commun. 17, 4037. 10.1038/s41467-026-70071-5 41839876 PMC13139411

[B46] Watson J. L. Juergens D. Bennett N. R. Trippe B. L. Yim J. Eisenach H. E. (2023). *De novo* design of protein structure and function with RFdiffusion. Nature 620, 1089–1100. 10.1038/s41586-023-06415-8 PMC10468394 37433327

[B47] Xiao Y. Sun S. Zheng N. Zhao J. Li X. Xu J. (2025). Development of PDAC diagnosis and prognosis evaluation models based on machine learning. BMC Cancer 25, 512. 10.1186/s12885-025-13929-z 40114140 PMC11924714

[B48] Yang J. Pan H. Sekimata K. Hwang C. Kulkarni A. Thomas H. (2025). A new BMP type 1 receptor kinase inhibitor for safe and efficient oral treatment to prevent genetically induced heterotopic ossification in mice. Bone 199, 117565. 10.1016/j.bone.2025.117565 40516669 PMC12332771

[B49] Zhang Z. Guan Q. Tian Y. Shao X. Zhao P. Huang L. (2023). Integrated bioinformatics analysis for the identification of idiopathic pulmonary fibrosis–related genes and potential therapeutic drugs. BMC Pulm. Med. 23, 373. 10.1186/s12890-023-02678-z 37794454 PMC10552267

